# Biochanin A enhances type H vessel formation and improves epiphysis deformities following ischemic osteonecrosis in juvenile mouse

**DOI:** 10.3389/fnut.2025.1583539

**Published:** 2025-07-02

**Authors:** Qian Huang, Yuchen Zhang, Shengping Tang, Xinda Zheng, Boxiang Li, Yun Liu, Xiaofei Ding, Jinmin Zhao, Qian Liu, Shijie Liao

**Affiliations:** ^1^Guangxi Key Laboratory of Regenerative Medicine, Department of Trauma Orthopedic and Hand Surgery, The First Affiliated Hospital of Guangxi Medical University, Nanning, China; ^2^Department of Orthopedics, Affiliated Minzu Hospital of Guangxi Medical University, Nanning, China; ^3^Department of Spine and Osteopathic Surgery, The First Affiliated Hospital of Guangxi Medical University, Nanning, China

**Keywords:** Legg–Calvé–Perthes disease, biochanin A, type H vessel, angiogenesis, PDGF-BB

## Abstract

Legg–Calvé–Perthes disease (LCPD) ranks as one of the most severe hip conditions that can lead to permanent deformity of the femoral head in children. Despite its severity, no effective pharmacological treatments are currently available, highlighting the urgent need for novel therapeutic agents. And platelet-derived growth factor-BB (PDGF-BB), a vital biological macromolecule, plays a critical role in vascular remodeling and bone regeneration, thereby establishing itself as a crucial drug target for activating bone repair. In this study, we demonstrate that biochanin A (BCA), a soybean-derived isoflavone, significantly prevents epiphyseal collapse and promotes bone regeneration in a juvenile ischemic osteonecrosis (JIO) model. Mechanistically, BCA enhances the formation of type H vessels in bone by indirectly facilitating the interaction between osteoclast precursors and endothelial cells, thereby accelerating bone repair in JIO. Specifically, BCA suppresses the differentiation of mature osteoclasts, expands the population of osteoclast precursors, and stimulates the secretion of PDGF-BB, which in turn promotes type H vessels angiogenesis. Our findings highlight the potential of BCA as a promising therapeutic candidate for the treatment of LCPD.

## 1 Introduction

Legg–Calvé–Perthes disease (LCPD) is an idiopathic avascular necrosis of the epiphysis of the femoral head, most commonly diagnosed in children aged 4 to 12 years (1). As one of the most prevalent hip disorders in children, LCPD can eventuate irreversible femoral head deformities and may predispose patients to early-onset osteoarthritis (2). Over the past five decades, treatment for LCPD has predominantly relied on surgical containment methods (3). However, in older children (≥8 years) or those with advanced stages, the curative effect for containment therapy is often poor (4). Furthermore, significant challenges related to patient compliance, as well as physical and psychological difficulties, limit the effectiveness and feasibility of conservative treatments (5). At present, no pharmacological therapies are available for LCPD, underscoring an urgent need to identify potential therapeutic agents (6).

Platelet-derived growth factor BB (PDGF-BB) plays a pivotal role in regulating bone physiology and pathology through its multifaceted biological functions (7). It acts as a key modulator of osteoblast proliferation and differentiation, while also promoting the recruitment and activation of osteoclast precursors, thereby influencing bone remodeling dynamics (8). In addition to its involvement in normal bone homeostasis, PDGF-BB contributes to the repair of skeletal injuries by stimulating angiogenesis and enhancing mesenchymal stem cell migration to sites of damage (9, 10). Dysregulated PDGF-BB signaling has been implicated in pathological conditions such as osteoporosis and bone metastases, highlighting its dual role in bone health and disease (11). These findings underscore the potential of targeting PDGF-BB as a therapeutic strategy for bone-related disorders, emphasizing the need for further research into its precise mechanisms of action.

Bioactive natural compounds have gained attention as promising candidates for bone repair drugs (12). Biochanin A (BCA) is an isoflavonoid found in natural products (chemical structure: 5,7-dihydroxy-4′-methoxyisoflavone, molecular formula C_16_H_12_O_5_, MW 284.26 g/mol), with naringenin as its precursor (13, 14). *In vivo*, BCA is metabolized into its demethylated isomer, daidzein, and genistein. Like most isoflavonoids, it exhibits extensive first-pass metabolism and a high volume of distribution (14). BCA can be isolated from red clover, chickpeas, and soybeans (15), exhibits anti-inflammatory, antioxidant, and anticancer effects, along with notable therapeutic potential in skeletal system disorders (16–18). In a double-blind, randomized, placebo-controlled trial involving 177 postmenopausal women aged 49 to 65, BCA supplementation over 12 months was found to reduce lumbar bone density loss (19). Subsequent research demonstrated that BCA, acting as a phytoestrogen, mitigates osteoporosis in ovariectomized rats by activating estrogen receptors and downstream signaling pathways (20). Additionally, BCA has been shown to alleviate osteoarthritis in mouse models by exerting anti-inflammatory, antioxidant, and iron overload-resistant effects (21, 22). Building on BCA’s anti-inflammatory properties, our previous study found that BCA mitigates inflammatory osteolysis by inhibiting excessive osteoclast activation (23). Collectively, the above-mentioned evidence highlights BCA as a promising candidate for accelerating the healing process in LCPD, though its specific effects on its pathology remain unclear.

To investigate BCA’s therapeutic potential for LCPD, we employed the juvenile ischemic osteonecrosis (JIO) mouse model, a widely recognized model for studying LCPD mechanisms and evaluating drug efficacy (24–27). Through this model, we observed that: (1) *in vivo*, BCA reduces bone loss in the epiphyseal regions and improves epiphyseal deformities by promoting type H vessel formation; (2) *in vitro*, BCA enhances endothelial cell angiogenesis by facilitating osteoclast precursors accumulation and stimulating PDGF-BB production. This study aims to evaluate whether BCA can alleviate deformities associated with epiphyseal ischemic necrosis in the JIO model.

## 2 Method and materials

### 2.1 JIO model establishment and BCA intervention

The selection of the murine distal femoral epiphysis over the proximal femoral epiphysis was based on three key reasons. First, the distal femur is one of the largest skeletal segments in mice, with an epiphyseal volume nearly four times that of the proximal femoral epiphysis, providing a greater tissue sample for experimental analysis. Second, its more superficial anatomical location enhances surgical accessibility compared to the proximal epiphysis. This advantage enables precise microsurgical induction of ischemic osteonecrosis while reducing complications such as hip dislocation, which is common in proximal approaches requiring extensive soft tissue dissection. Third, a key biological distinction further supports this choice: in mice, the proximal femoral epiphysis (femoral head) ossifies through direct remodeling of calcified cartilage via metaphyseal vascular invasion, differing from the human femoral head, which develops through secondary ossification centers (28). In contrast, the murine distal femoral epiphysis undergoes endochondral ossification via secondary ossification center formation, closely mirroring the developmental process of the human femoral head (28).

All animal procedures were approved by the Animal Care & Welfare Committee of Guangxi Medical University (Approval Number: 202311008). Since LCPD predominantly affects male children (29), we selected 4-week-old male C57BL/6 mice for modeling. Mice were obtained from the Guangxi Medical University Laboratory Animal Center. The JIO model was created following established methods (27). Under anesthesia induced by tribromoethanol, ischemic necrosis of the distal femoral epiphysis was achieved through microsurgical coagulation of four key blood vessels supplying the left femoral epiphysis. For vascular cauterization, two surgical approaches were used: a posterior-medial incision for the popliteal vessels and a medial patellar incision to target the lateral, medial, and central genicular arteries. Postoperative sodium penicillin (18 mL/kg day) was administered via intramuscular injection for 3 days to prevent infection, and carprofen (5 mg/kg every 12 h) was given subcutaneously for pain management for 2 days.

A total of 14 mice were subjected to modeling and randomly divided into two groups: a JIO model group receiving 1 ‰ DMSO-PBS (JIO group, *n* = 7) and a JIO model group treated with BCA (JIO + BCA group, *n* = 7). In our study, the control group refers to the contralateral (right) femur of the same JIO model mice. The BCA purchased from Chengdu Must Bio-Technology (A0419) and dissolved in 1 ‰ DMSO-PBS. The BCA dosage used in this study (5 mg/kg b.wt) was selected based on our and others’ previous published works (20, 23). The BCA intervention involved intraperitoneal injection at a concentration of 5 mg/kg every 2 ays, following our previous protocol (23). BCA treatment commenced on postoperative day 7. After 5 weeks of intervention, femurs were collected for Micro-CT analysis (*n* = 4) and immunostaining (*n* = 3). No adverse events were observed during the animal experiment.

### 2.2 Micro-CT scanning and analysis of bone tissue

After the mice were euthanized, bilateral femurs were collected and fixed in 4% paraformaldehyde (Biosharp). Femurs were immersed in 75% ethanol for storage. Subsequently, the samples were scanned by a Skyscan1176 micro-CT scanner (Buker) with the following parameters: AI = 0.5 mm, voltage = 65 kV, current = 439 μA, and resolution = 9 μm. The raw images were reconstructed using DataViewer (version: 1.5.4.0, Buker) and CTvox software (version: 3.3.0 r1403, Buker). Bone trabecular parameters in the zone of interest were analyzed with CTAn software (version: 1.15.2.2, Buker). For the distal femoral epiphysis, the zone of interest was defined from the fusion point of the medial and lateral condyles of the femur, extending 50 layers upward to include only epiphyseal trabecular bone.

### 2.3 Immunostaining for bone tissue

After dissection, the lower limbs were collected, and femurs were isolated with surrounding muscles and soft tissues carefully removed. The bones were fixed in 4% paraformaldehyde for 4 h, decalcified in 10 mL EDTA decalcifying solution (E1171, Solarbio) at 4°C for 48 h, and rinsed five times in PBS. Samples were then immersed in a cryoprotective solution containing 200 g sucrose (S8271, Solarbio) and 20 g polyvinylpyrrolidone (PVP) (P8290, Solarbio) in PBS 24 h at 4°C. For embedding bone tissue, a few drops of embedding agent should be added to the PVC embedding box (0203-0007, CITOTEST). This agent is prepared by dissolving 8 g of gelatin (V900863, Sigma-Aldrich), 2 g of PVP, and 20 g of sucrose in 80 mL of PBS, adjusting the volume to 100 mL, and incubating at 65°C until the solution becomes clear. The bone sample should be carefully placed in the embedding agent, taking care to avoid any bubbles, and the embedding box should then be filled completely with the agent. The sample should be stood at room temperature for 30 min to allow the embedding agent to solidify completely, before transferring to a −80°C freezer. The frozen bone samples were first removed from storage at −80°C and then placed in a cryostat (Leica CM1950) for pre-cooling for 60 min. To obtain comprehensive structural views of the bone tissue, sections were prepared in a longitudinal orientation. Freshly precooled blades were used to section the tissues at −23°C, resulting in 50 μm-thick bone sections. Following sectioning, the samples were mounted onto room-temperature glass slides, facilitating optimal adhesion by leveraging the temperature differential between the sections and slides. The mounted sections were then air-dried at room temperature for 30 min and subsequently stored at −20°C.

For staining, slides stored at −20°C were unfroze at room temperature for 20 min. Embedding medium was then removed, and a hydrophobic barrier was drawn around the tissue section by a Pap pen. Next, 200 μL of PBS were added to the sections, which were incubated at room temperature for 5 min to achieve complete hydration. Permeabilization was performed by incubating the sections in 0.3% TritonX-100 (T8200, Solarbio) for 20 min at room temperature. Following permeabilization, sections were blocked with 5% animal serum (matching the host species of the secondary antibody) for 30 min to avoid nonspecific fluorescence. After blocking, the primary antibody was incubated in the dark, either overnight at 4°C or for 2 h at room temperature. After the incubation of primary antibody, the sections were washed with PBS five times, for 3 min each. Then the secondary antibody was incubated at room temperature for 75 min. Following secondary antibody incubation, sections were again washed with PBS five times, for 3 min each. To counterstain nuclei, DAPI (D9542, Sigma-Aldrich, 1:500) diluted in PBS was incubated at room temperature for 30 min. Afterward, sections were washed with PBS five more times, for 3 min each, to ensure thorough removal of excess DAPI. After aspirating all PBS, 100 μL of Fluoromount-G^®^ (0100-01, SouthernBiotech) was added to the slide, which was then overlapped with a cover glass, carefully avoiding air bubble formation. The edges of the coverslip were sealed with nail polish to secure the preparation and protect it from light during storage at 4°C. Slides were captured by a confocal laser-scanning microscopy (STELLARIS5, Leica) within 48 h to prevent signal degradation. Details of the primary and secondary antibodies used in this procedure are provided in Supplementary Table 1.

### 2.4 HUVEC culture and cell migration assay

Human umbilical vein endothelial cells (HUVECs, CL-0675) were obtained from Pricella (Wuhan, China) and cultured in specialized HUVEC medium (CM-0675, Pricella) at 37°C in a 5% CO_2_ incubator. Upon reaching 90% confluence, cells were treated with EDTA-trypsin and seeded at a density of 1.0 × 10^4^ cells per Culture-Insert 4 Well (80466, ibidi). After removing the inserts and washing with PBS, cells were treated with either 16 μM BCA or control medium in serum-free RPMI-1640 medium (PM150110, Pricella). Images were captured at 0 and 12 h by a Cytation 5 microscope (BioTek), and wound healing was analyzed using Fiji software (NIH, Bethesda, MD), and the wound healing rate was assessed using the following formula:


$$\text{Wound healing rate}=(W_{0}-W_{h})/W_{0}\times 100\%$$

where *W*_0_ is the initial scratch region at 0 h, and *W*_h_ is the scratch region after 12 h.

### 2.5 Bone marrow-derived macrophage extraction and osteoclast differentiation

Bone marrow-derived macrophage (BMMs) were extracted from the femurs of C57BL/6 mice. BMMs (6.0 × 10^3^/well) were cultured in *α*-MEM medium (12561-056, Gibco) with 25 ng/mL M-CSF (R&D, 416-ML-050/CF), 10% fetal bovine serum (10091–148, Gibco), and 1% penicillin–streptomycin (15070063, Gibco) at 37°C and 5% CO_2_. Experimental groups included negative control, a positive control (50 ng/mL RANKL), and various BCA concentrations (2, 4, 8, 16 μM) with RANKL. Tartrate-resistant acid phosphatase (TRAP)-positive cells with three or more nuclei were classified as mature osteoclasts, while with fewer than three nuclei were categorized as osteoclast precursors. The numbers of osteoclasts and osteoclast precursors were quantified using Fiji software.

### 2.6 Immunostaining for cell

BMMs were seeded at a density of 1.0 × 10^5^/confocal culture dish, differentiated using 25 ng/mL M-CSF and 50 ng/mL RANKL, and processed for immunofluorescence. Cells were fixed, permeabilized, blocked with 5% BSA (4240GR500, BioFroxx), and incubated with primary and secondary antibodies sequentially. Images were captured by a confocal laser-scanning microscopy, with all antibody details provided in Supplementary Table 1.

### 2.7 Conditioned media preparation and HUVEC intervention

BMMs were seeded at a density of 1.0 × 10^5^ cells/well and cultured overnight. The cells were divided into the following groups for treatment: positive control group (25 ng/mL M-CSF + 50 ng/mL RANKL), and BCA group (16 μM BCA + 50 ng/mL RANKL). The medium was refreshed every 2 days, and the conditioned medium (CM) was collected on days 5–7. The CM from each group was centrifuged at 3,000 × g for 10 min, and the supernatant was stored at −80°C for subsequent experiments.

HUVECs were seeded with a density of 8.0 × 10^3^/well of a scratch assay insert (81176, Culture-Insert 2 Well, ibidi). When the cells were full of inserts, the inserts were removed, and the cells were washed once with PBS. The collected CM was added to serum-free medium, and the cell migration area was captured after 12 h by a Cytation 5 microscope. The scratch areas were analyzed using Fiji software, and the wound healing rate was calculated using the above-mentioned formula.

### 2.8 CCK-8 cell viability assay

The CCK-8 assay (HY-K0301, MCE) was used to assess the cytotoxicity of BCA on BMM and HUVEC cells. Cells were seeded at 6.0 × 10^3^ cells/well, treated with BCA (0, 2, 4, 8, 16 μM), incubated for 48 h, and analyzed for absorbance at 450 nm by a multifunctional microplate reader (Tristar2LB 942, Berthold).

### 2.9 Statistical analysis

All data are presented as mean ± standard deviation (SD). Unpaired *t*-tests were used for two-group comparisons, and one-way ANOVA for three or more groups. Statistical analysis was performed using GraphPad Prism 8.0.1 (GraphPad Software, San Diego, United States), with *p*-values <0.05 considered statistically significant.

## 3 Results

### 3.1 BCA improves epiphysis deformities in JIO mice

**Figure 1:**
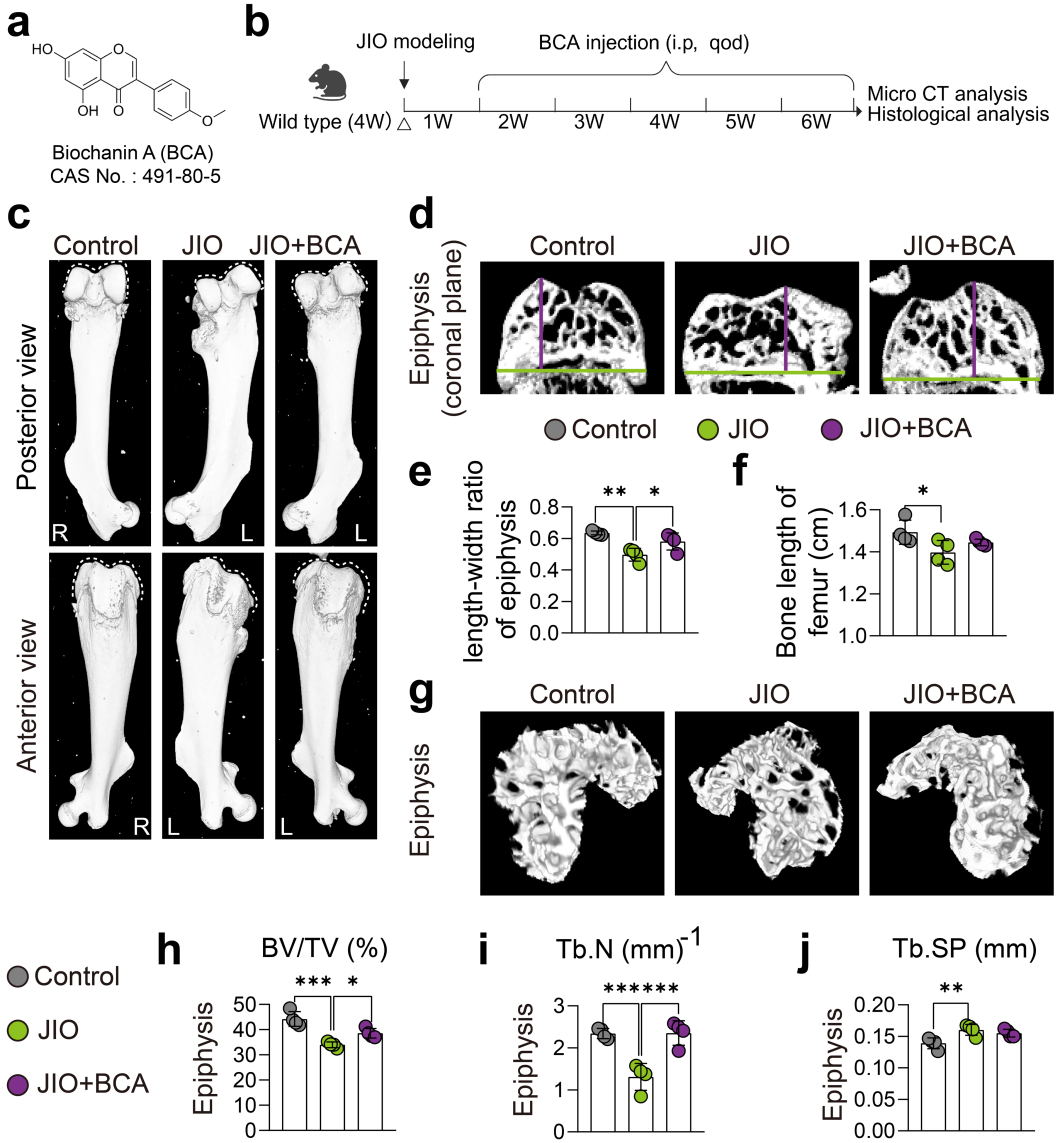
BCA improves epiphysis deformities in JIO mice. **(a)** Chemical structure of BCA. **(b)** Schematic representation of the intervention protocol using BCA in juvenile ischemic osteonecrosis (JIO) mice. **(c)** Gross observation from anterior and posterior views showing that BCA improves the collapse deformity of the distal femoral epiphysis in JIO mice. **(d–f)** Coronal sections of the distal femoral epiphysis demonstrate that BCA significantly increases the length-to-width ratio of the epiphysis, indicating its potential to ameliorate the collapse deformity. **(g)** Micro-CT reconstructed images of the epiphysis further confirm that BCA intervention mitigates bone loss post-ischemia in JIO mice. **(h–j)** Statistical analysis of bone volume fraction (BV/TV), trabecular number (Tb.N), and trabecular spacing (Tb.Sp). Data are presented as mean ± *SD*. *p < 0.05, **p < 0.01, ***p < 0.001 and ****p < 0.0001.

If untreated, LCPD may cause severe deformity of the femoral head, potentially leading to osteoarthritis (1). To investigate the effects of BCA on epiphyseal deformities in the JIO model, we conducted micro-CT imaging, revealing that BCA treatment effectively mitigated femoral epiphyseal deformities compared to the untreated model group (Figures 1a,b). Additionally, BCA treatment significantly restored the length-to-width ratio of the epiphysis (Figures 1c–e) and moderately increased femoral length, though it did not reach normal levels (Figure 1f). Compared with untreated JIO mice, BCA-treated mice exhibited increased bone volume fraction (BV/TV) in epiphyseal regions, along with increased trabecular number (Tb.N) (Figures 1g–j), indicating that BCA enhances bone mass in necrotic regions and helps restore normal bone architecture. It is noteworthy that mice treated with BCA did not exhibit any signs of hepatotoxicity or nephrotoxicity (Appendix Figures 1, 2).

### 3.2 BCA induces type H vessel formation in JIO mice

**Figure 2:**
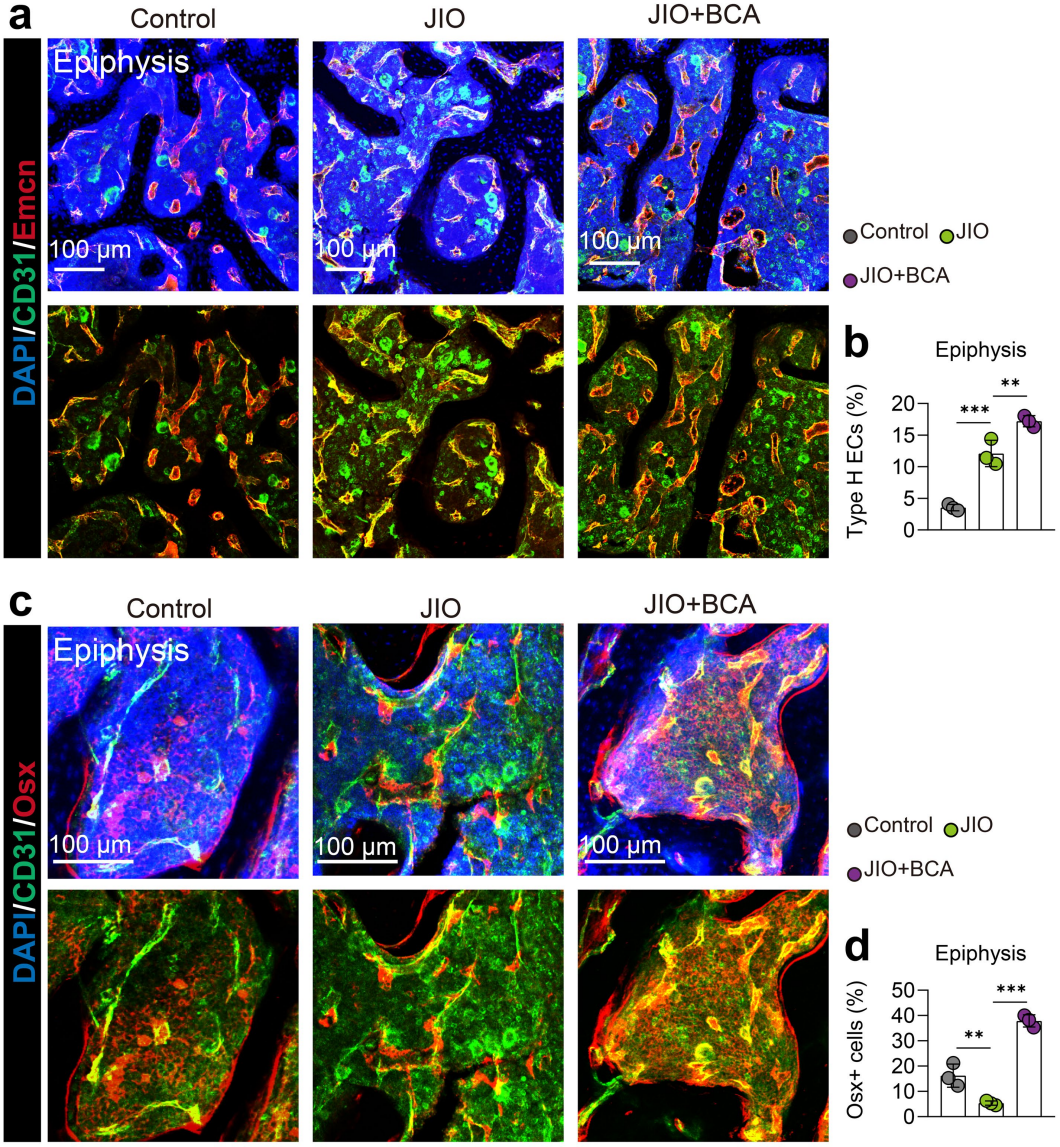
BCA induces type H vessel formation in epiphysis of JIO mice. **(a)** Representative immunofluorescence images of CD31/Emcn vessels (type H vessels) in the epiphysis of distal femur of control, JIO, and BCA-treated JIO mice. **(b)** Statistical analyses of the number of type H endothelial cells in the distal femoral epiphysis. **(c)** Representative immunofluorescence images of Osx^+^ cells in the epiphysis of distal femur of control, JIO, and BCA-treated JIO mice. **(d)** Statistical analyses of the number of Osx^+^ cells in the distal femoral epiphysis. Data are presented as mean ± *SD*. *p < 0.05, **p < 0.01, ***p < 0.001 and ****p < 0.0001.

The pathogenesis of LCPD primarily stems from disruption of epiphyseal blood supply, making revascularization essential for bone repair and healing (30). Therefore, we further assessed the impact of BCA on type H vessel formation in the epiphysis of JIO model. Immunofluorescence staining demonstrated an increased abundance of type H vessels in the epiphysis during the reconstitution phase (6 weeks post-induction) compared to controls, suggesting active bone formation within the JIO model. BCA treatment further significantly increased type H vessel (Emcn^hi^ CD31^hi^ endothelial) density in the epiphysis of JIO mice (Figures 2a,b). Simultaneously, BCA markedly augmented the population of Osx^+^ cells, aligning with the perspective of type H vessels coupling osteogenesis (Figures 2c,d). Given the critical role of type H vessels in bone repair (31), these results suggest that BCA promotes bone repair by enhancing type H vessel formation in the JIO model.

### 3.3 BCA inhibits endothelial angiogenesis *in vitro*

**Figure 3:**
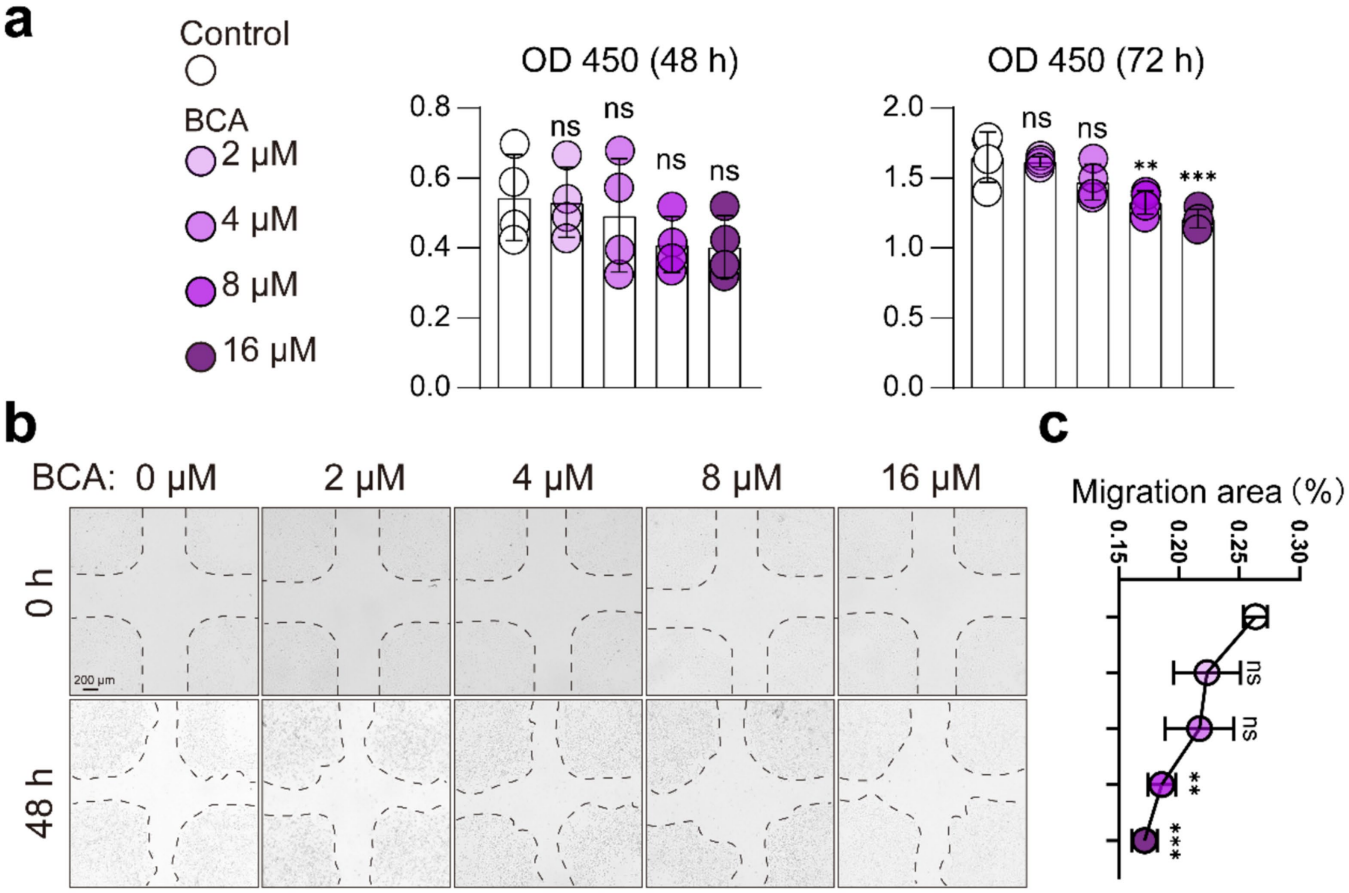
BCA inhibits endothelial cell viability and migration. **(a)** Effects of various concentrations of BCA (0, 2, 4, 8, and 16 μM) on the viability of HUVECs. **(b,c)** Effects of various concentrations of BCA (0, 2, 4, 8, and 16 μM) on the migration ability of HUVECs, with corresponding statistical analyses of the migration area. Data are presented as mean ± *SD*. *p < 0.05, **p < 0.01, ***p < 0.001 and ****p < 0.0001.

Previous studies have indicated that BCA inhibits angiogenesis (32, 33), which contrasts with our *in vivo* findings in the JIO model. To explore the underlying mechanism, we evaluated BCA’s effects on endothelial cells (HUVECs) *in vitro*. The CCK8 assay showed that BCA (8 μM, 16 μM) significantly suppressed HUVEC viability after 72 h (Figure 3a), and migration assays confirmed that BCA reduced HUVEC migratory capacity (Figures 3b,c). These results align with previous studies, indicating that BCA indeed exerts inhibitory effects on vascular function *in vitro* (32, 33).

### 3.4 BCA suppresses osteoclast differentiation and induces osteoclast precursor generation

Our previous research demonstrated that BCA mitigates inflammatory osteolysis by inhibiting excessive osteoclast activation (23). Additionally, studies have shown that inhibiting osteoclast differentiation enlarges the pool of osteoclast precursors, enabling them to secrete factors such as PDGF-BB, which supports type H vessel angiogenesis (9, 10, 34). We hypothesized that BCA may activate type H vessel formation in JIO mice by promoting coupling between osteoclast precursors and type H endothelium, rather than by directly stimulating endothelial cells.

**Figure 4:**
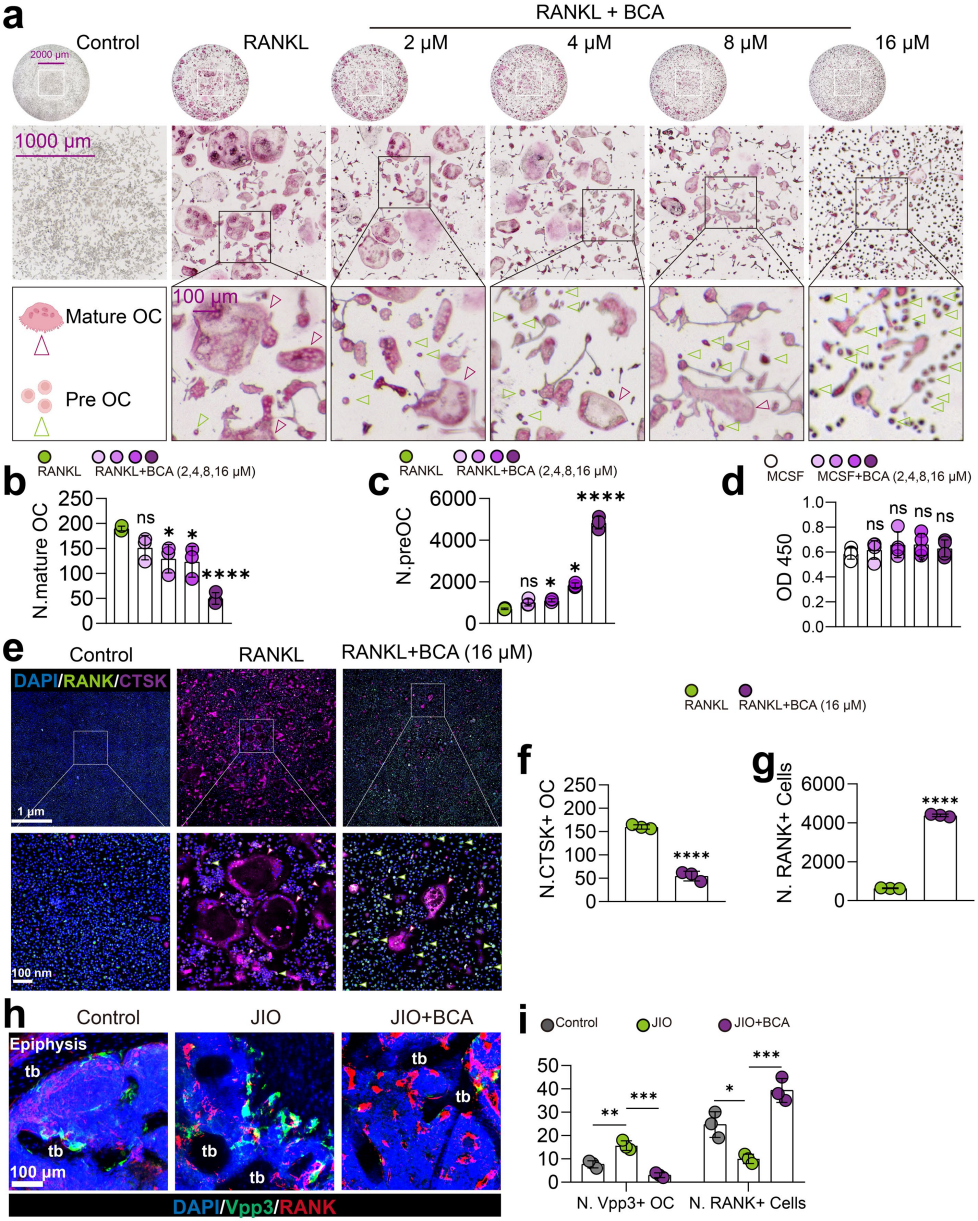
BCA restrains osteoclast differentiation and increases the number of osteoclast precursor cells. **(a–c)** Effects of various concentrations of BCA (0, 2, 4, 8, and 16 μM) on the differentiation of BMMs into osteoclasts, with corresponding statistical analyses of the number of mature osteoclasts and osteoclast precursor cells. **(d)** Effects of various concentrations of BCA (0, 2, 4, 8, and 16 μM) on the viability of BMMs. **(e–g)** Representative immunofluorescence images of RANK^+^ cells (osteoclast precursor cells) and CTSK^+^ cells (mature osteoclasts) after BCA (16 μM) intervention *in vitro*, with corresponding statistical analyses of the number of RANK^+^ and CTSK^+^ cells. **(h,i)** Representative immunofluorescence images of Vpp3/RANK staining in the distal femoral epiphysis of control, JIO, and BCA-treated JIO mice, where RANK^+^ cells represent osteoclast precursor cells and Vpp3^+^ cells represent mature osteoclasts. Data are presented as mean ± *SD*. *p < 0.05, **p < 0.01, ***p < 0.001 and ****p < 0.0001.

To test this hypothesis, we assessed BCA’s effects on bone marrow macrophages (BMMs). CCK-8 assays indicated that BCA did not affect BMM viability within the concentration range of 16 μM (Figure 4d). And BCA inhibited RANKL-induced osteoclast formation in a concentration-dependent manner starting from 4 μM, consistent with previous findings (Figures 4a,b) (23). More importantly, BCA notably grew the population of osteoclast precursor cells (Figure 4c). Immunofluorescence analysis further revealed that BCA significantly increased CTSK^+^ RANK^+^ osteoclast precursor numbers under RANKL stimulation (Figures 4e–g). Additionally, we verified *in vivo* that BCA induces osteoclast precursor generation in the epiphyseal regions of the JIO model (Figures 4h,i).

### 3.5 BCA indirectly enhances the migration of endothelial cells by facilitating the expression of PDGFBB in osteoclast precursor cells

**Figure 5:**
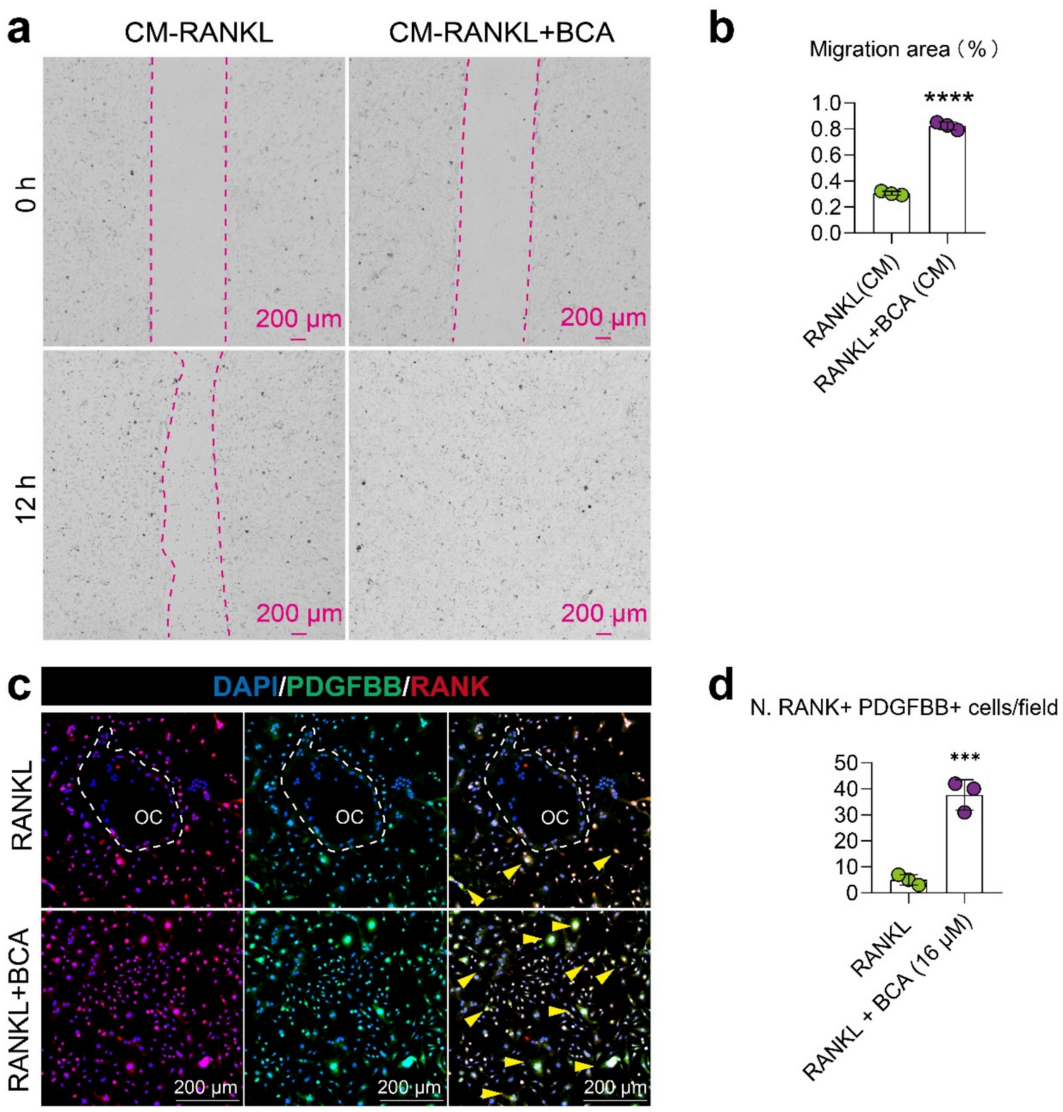
BCA indirectly enhances the migration of endothelial cells by facilitating the expression of PDGF-BB in osteoclast precursor cells. **(a,b)** Representative images and corresponding statistical graphs depicting the effect of conditioned media from BMMs treated with RANKL alone and in combination with BCA on the migration ability of endothelial cells. The conditioned media were collected from BMMs under RANKL intervention with or without BCA treatment. **(c,d)** Representative images of double immunofluorescence staining for RANK/PDGF-BB in BMMs differentiated into osteoclasts after *in vitro* BCA (16 μM) intervention, along with statistical analysis of the number of RANK^+^ PDGF-BB^+^ cells. Data are presented as mean ± *SD*. *p < 0.05, **p < 0.01, ***p < 0.001 and ****p < 0.0001.

To further investigate whether the promotion of type H vessel formation in JIO mice by BCA is mediated through its effects on osteoclast precursor cells, we conducted coculture experiments using conditioned media from osteoclasts pre- and post-BCA intervention with endothelial cells. We observed that the conditioned media from BCA-treated osteoclasts significantly promoted the migration of endothelial cells (Figures 5a,b), indicating that BCA indirectly enhances angiogenesis through osteoclast precursor-H vessel coupling. Previous literature has shown that osteoclast precursor cell coupling to type H vessels depends on the secretion of PDGF-BB by these cells (9, 10). Subsequently, we employed cellular immunofluorescence staining to demonstrate that BCA significantly increased the number of PDGF-BB^+^ osteoclast precursor cells (Figures 5c,d). Therefore, our experimental results suggest that BCA indirectly promotes the activity of vascular endothelial cells by facilitating the expression of PDGF-BB in osteoclast precursor cells.

### 3.6 BCA increases the number of osteoclast precursor cells and induces PDGF-BB production in JIO mice

**Figure 6:**
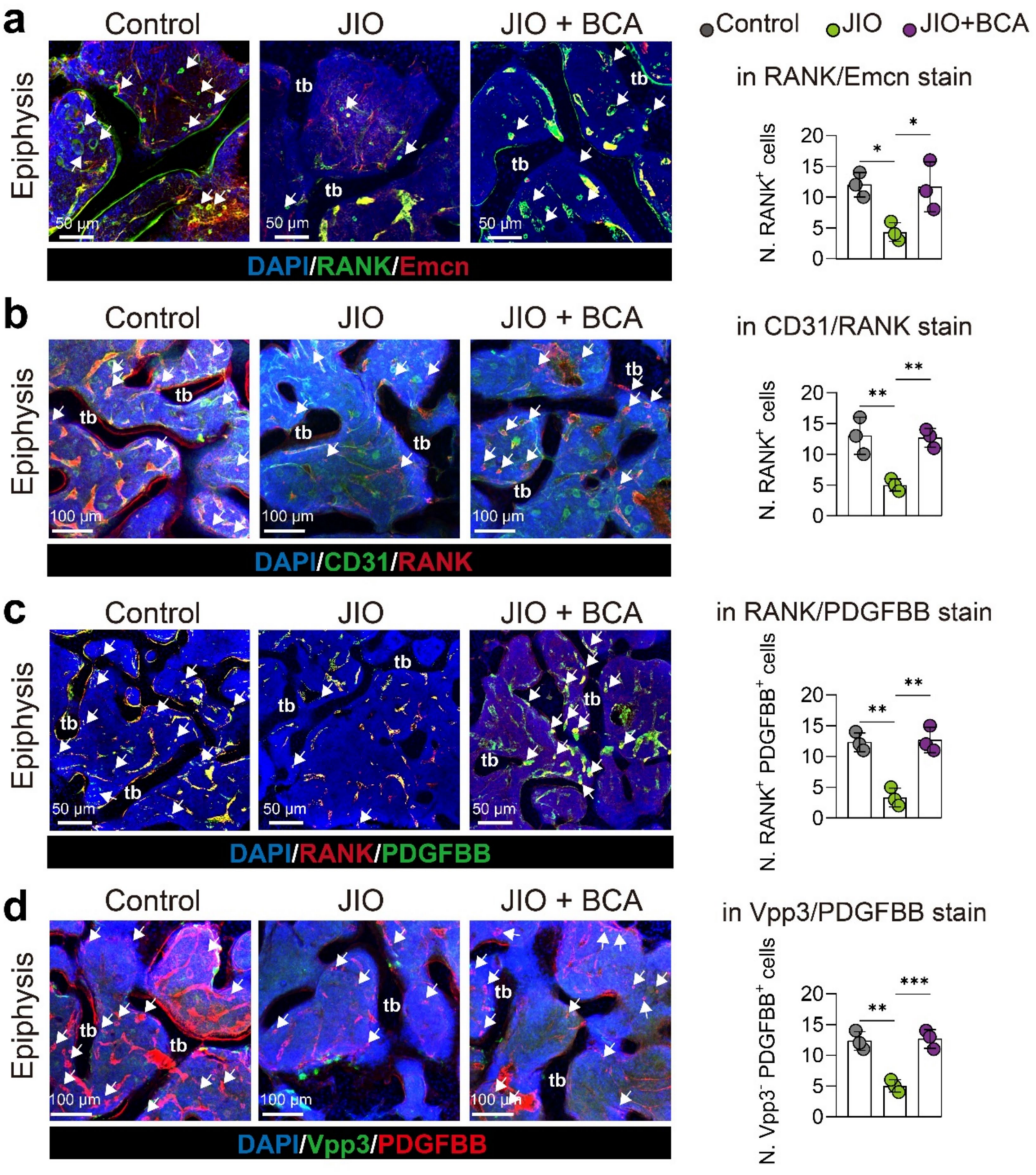
BCA increases the number of osteoclast precursor cells and induces PDGF-BB production in epiphysis of JIO mice. **(a)** Representative immunofluorescence staining images for RANK/Emcn stain with statistical analysis of the number of RANK^+^ cells. White arrows indicate RANK^+^ osteoclast precursor cells. **(b)** Representative immunofluorescence staining images for CD31/RANK stain with statistical analysis of the number of RANK^+^ cells. White arrows indicate RANK^+^ osteoclast precursor cells. **(c)** Representative immunofluorescence staining images for RANK/PDGF-BB stain with statistical analysis of the number of RANK^+^ PDGF-BB^+^ cells in RANK/PDGF-BB staining. White arrows point to RANK^+^ PDGF-BB^+^ osteoclast precursor cells. **(d)** Representative immunofluorescence staining images for Vpp3/PDGF-BB stain with statistical analysis of the number of Vpp3-negative but PDGF-BB^+^ cells (Vpp3^−^ PDGF-BB^+^). White arrows indicate Vpp3-negative but PDGF-BB^+^ osteoclast precursor cells. Data are presented as mean ± *SD*. *p < 0.05, **p < 0.01, ***p < 0.001 and ****p < 0.0001.

Finally, we validated *in vivo* that BCA intervention promotes the generation of RANK^+^ osteoclast precursor cells through fluorescent staining of bone tissue sections. Notably, the number of these RANK^+^ osteoclast precursor cells positively correlated with the number of Emcn^+^ and CD31^+^ endothelial cells, suggesting their coupling relationship with type H vessels (Figures 6a,b). Previous literature has indicated that endothelial cells are also a source of PDGF-BB, whereas PDGF-BB is expressed only in low levels in mature osteoclasts (11). Therefore, we examined the expression of PDGF-BB in RANK^+^ cells (representing osteoclast precursor cells) and Vpp3^+^ cells (representing mature osteoclasts). PDGF-BB was predominantly enriched in RANK^+^ cells and less frequently observed in Vpp3^+^ cells (Figures 6c,d). Importantly, BCA administration significantly increased the number of RANK^+^ PDGF-BB^+^ cells (Figure 6c). Consequently, our experimental results also demonstrate that BCA exhibits an indirect angiogenic effect *in vivo* that is consistent with the findings from *in vitro* experiments.

## 4 Discussion

LCPD is a self-limiting condition characterized by initial stages of avascular necrosis and fragmentation, followed by a prolonged bone regeneration phase lasting 2 to 4 years (35). In its early stages, the epiphyseal structure is weakened, and hip joint weight-bearing can induce epiphyseal deformities, although these remain reversible. However, as the disease progresses into modified Waldenström stages IIb–IIIa, an imbalance emerges between extensive bone resorption and insufficient bone formation, with the immature woven bone failing to mature into dense, organized lamellar bone. This imbalance leads to irreversible epiphyseal deformities (29, 36). Consequently, the management of LCPD focuses on early intervention, aiming to preserve the spherical shape of the femoral head and reduce the risk of early-onset arthritis (37). Preventative treatments include non-surgical approaches such as weight-bearing relief, braces, and casting, as well as surgical strategies like proximal femoral osteotomies and combined surgical techniques (5). Despite these options, the efficacy of conservative treatments remains contentious (38, 39), particularly for adolescents with LCPD, where treatment success is often limited (40–42). This has led to growing interest in pharmacological approaches that could both expedite the natural course of LCPD and complement surgical interventions. Initial studies have investigated agents like MR16-1 (6), tocilizumab (26), strontium ranelate (43), ibandronate, and bone morphogenetic protein-2 (BMP-2) (44) in LCPD animal models.

Natural products are gaining recognition in drug discovery due to their varied sources, low toxicity, and multi-target mechanisms (45, 46). However, research on their application for LCPD is sparse. Our preliminary work identified biochanin A (BCA), a soy-derived isoflavone, as a candidate for preventing femoral head necrosis and collapse in young rats, primarily by ameliorating endothelial dysfunction (47, 48). In this study, we examined BCA’s impact on bone angiogenesis in a JIO mice model, focusing on its potential to stimulate type H vessel formation, a key driver of the self-limiting repair process in LCPD. Our results demonstrate that BCA significantly promotes type H vessel formation in epiphysis. Extensive research has established that type H vessels play a crucial role in skeletal development, bone degeneration, and fracture repair (31, 49–51). By coupling with osteogenesis, type H vessels facilitate fracture healing, while their reduction is a contributing factor to bone loss in osteoporosis (52). Both in mice and humans, the density of type H vessels declines with age, peaking in childhood, consistent with the peak incidence of LCPD (29, 51). Our unpublished findings further suggest that type H vessels are reactivated in the JIO model’s epiphysis, exceeding control levels at similar stages, underscoring their critical role in the self-repair mechanism of LCPD. This study confirms BCA’s potent angiogenic potential in enhancing bone regeneration.

Notably, BCA has shown anti-angiogenic properties *in vitro*, contradicting our *in vivo* findings. BCA has been reported to inhibit endothelial cell function and angiogenesis, thereby suppressing tumor growth and metastasis across various cancers (32, 53, 54). This apparent contradiction may be attributed to the unique characteristics of type H vessels, which are specifically associated with skeletal system angiogenesis, unlike angiogenesis in other organs (55). Endothelial cells exhibit tissue-specific heterogeneity (56). Notably, type H vessels in bone possess unique regulatory mechanisms distinct from those in other organs (57). For example, while notch signaling typically suppresses angiogenesis in various systems, it actively promotes both angiogenesis and osteogenesis in bone (50, 58). Due to the current inability to culture primary type H endothelial cells *in vitro*, we used HUVECs as a surrogate. Consistent with prior studies, BCA exhibited inhibitory effects on HUVECs at concentrations of 10.5 μM (59). Other reports have noted suppression at even higher concentrations (100 μM) (60), and sensitivity appears to differ by endothelial cell type (54, 59). Furthermore, bone vasculature, particularly type H vessels, is regulated by blood flow, and impaired perfusion has been shown to reduce their abundance and impair osteogenesis (61). While BCA is known to induce vasodilation in large arteries (33, 62, 63), its impact on bone blood flow and type H vessel regulation remains unclear and warrants further investigation.

Additionally, the bone marrow environment contains cell types, such as osteoclast precursors, that are scarce in extra-skeletal tissues. Prior studies have demonstrated that osteoclast precursors promote type H vessel formation by secreting PDGF-BB (9, 10), and PDGF-BB levels have been reported to decrease in the synovial fluid of early-stage LCPD patients (64). In our study, BCA was shown to expand the osteoclast precursor pool and enhance PDGF-BB secretion, inducing type H endothelial formation in the epiphyseal regions of the JIO model. Our previous research showed that BCA inhibits mature osteoclast formation by downregulating NF-κB and MAPK signaling pathways (23), likely leading to osteoclast precursor accumulation. The improvement of epiphyseal deformities in the JIO model observed with BCA may also stem from its inhibitory effect on excessive osteoclastic bone resorption. Collectively, these results suggest that BCA’s therapeutic effects in the JIO model arise from an indirect coupling between osteoclast precursors and type H endothelium, rather than a direct pro-angiogenic effect on endothelial cells. On the other hand, the improvement in epiphyseal morphology following BCA treatment may also attribute to its promotive effect on osteogenic differentiation. BCA has been shown to facilitate the differentiation of adipose-derived stem cells into osteoblasts while inhibiting adipogenesis (65), which may support trabecular reconstruction after epiphyseal osteonecrosis. However, the specific mechanisms underlying this effect remain to be elucidated.

This study has certain limitations. First, the optimal BCA dosage for LCPD treatment remains unknown; the concentration used here was based on effective doses in previous osteolysis studies (23). Second, we have not been able to evaluate the plasma concentration of BCA in the JIO mice to validate the systemic exposure of BCA. Third, the specific concentration of BCA reaching the epiphyseal necrotic area is uncertain. BCA treatment was administered continuously from the onset of necrosis through the epiphyseal healing phase. As vascularization progresses, the amount of BCA reaching the epiphyseal necrotic site may vary over time. According to our results, BCA may be a promising candidate for accelerating epiphyseal repair in LCPD patients, preventing excessive deformities. However, the low oral bioavailability of BCA limits its clinical translation potential (14, 66). Future studies should focus on exploring strategies to improve BCA’s solubility and bioavailability, such as developing suitable drug delivery systems, which hold promising prospects (67).

## 5 Conclusion

In conclusion, our study shows that BCA, a soy-derived isoflavone, effectively prevents epiphyseal collapse and promotes bone formation in the JIO model. Mechanistically, BCA supports type H vessel formation in bone by indirectly facilitating osteoclast precursor and endothelial cell coupling, thereby accelerating bone repair in LCPD. Specifically, BCA inhibits mature osteoclast formation, expands the osteoclast precursor pool, and enhances PDGF-BB secretion, indirectly promoting endothelial angiogenesis. These findings suggest that BCA has therapeutic potential for LCPD management.

## Data Availability

The original contributions presented in the study are included in the article/Supplementary material, further inquiries can be directed to the corresponding authors.
