# Supplementary material for: Biochanin A enhances type H vessel formation and improves epiphysis deformities following ischemic osteonecrosis in juvenile mouse
Source: Front Nutr. 2025 Jul 2;12:1583539. doi: 10.3389/fnut.2025.1583539 (PMC12263671; doi:10.3389/fnut.2025.1583539)
Supplement: Supplementary file 1 [file Image_1.pdf]

Appendices figures. 1

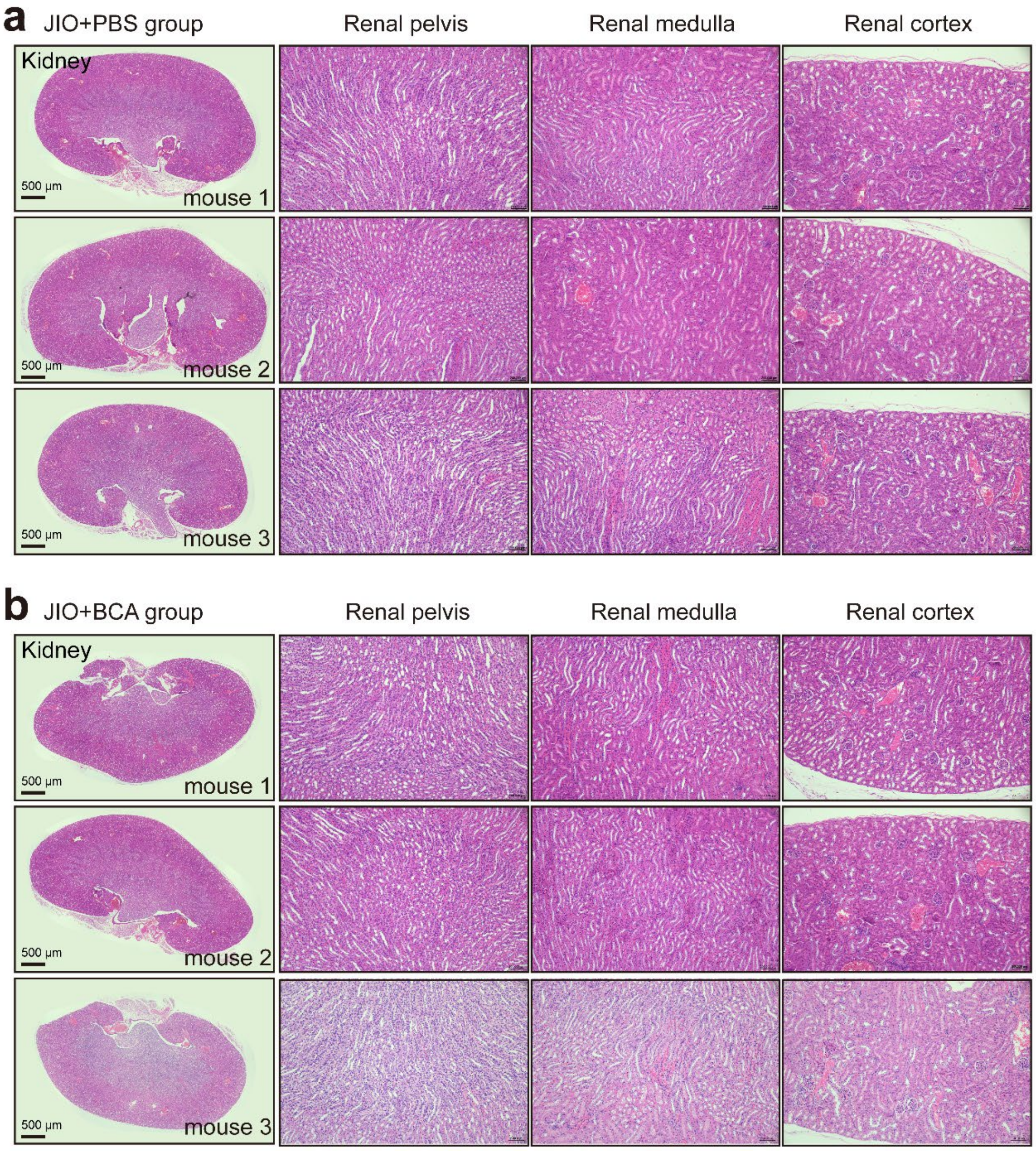

Appendices figures 1. BCA exhibits no nephrotoxic effects in young mice. (a) Representative hematoxylin and eosin (HE)-stained sections of the kidney from JIO mice in the PBS intervention group, with focal images of the renal pelvis, renal medulla, and renal cortex. (b) Representative HE-stained sections of the kidney from JIO mice in the BCA intervention group, with focal images of the renal pelvis, renal medulla, and renal cortex.

Appendices figures. 2

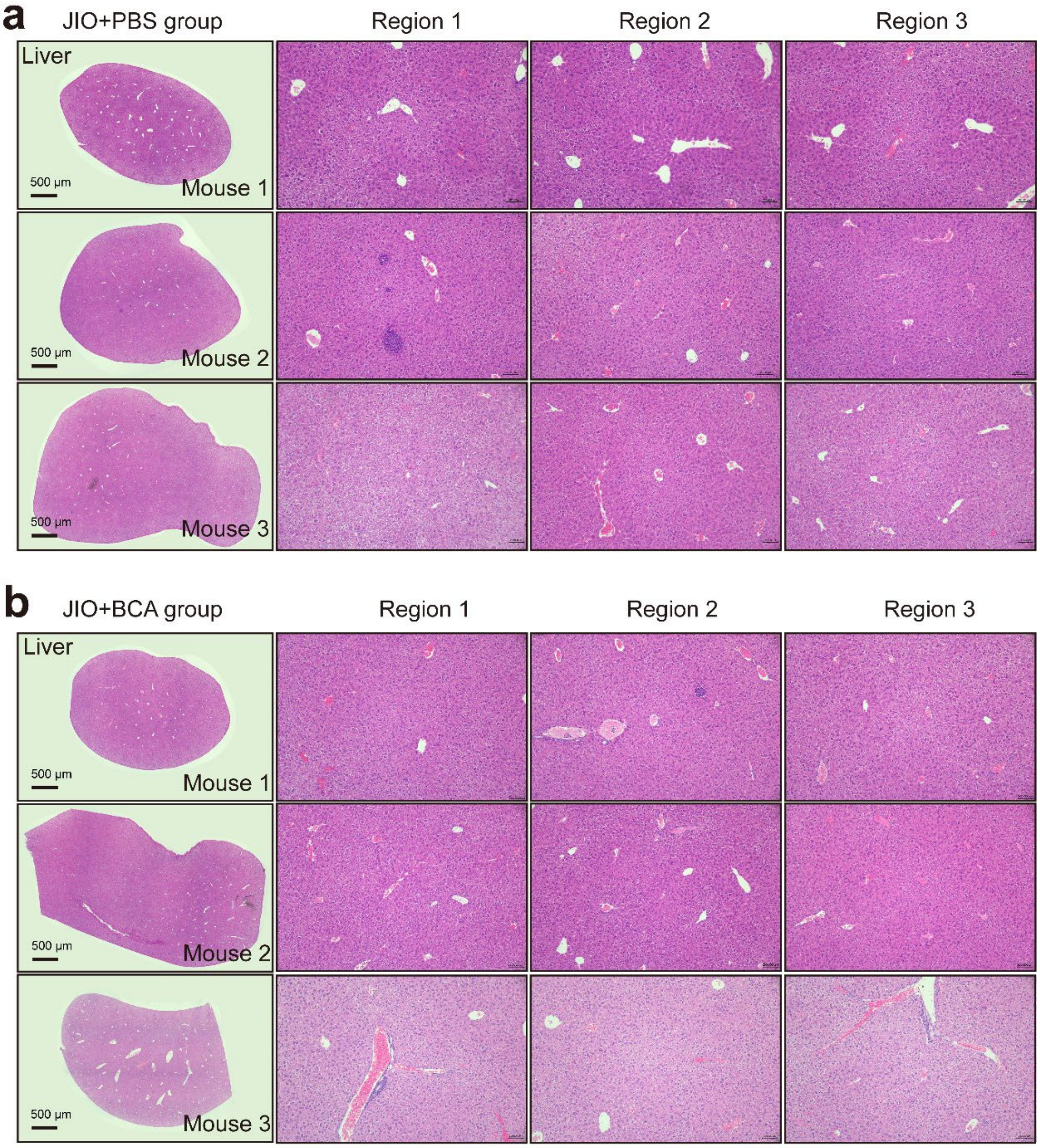

Appendices figures 2. BCA exhibits no hepatotoxicity in young mice. (a) Representative HE-stained sections of the liver from JIO mice in the PBS intervention group, with focal images of three regions. (b) Representative HE-stained sections of the liver from JIO mice in the BCA intervention group, with focal images of three regions.
